# Iran’s Journey Through Malaria: From Past Challenges to Future Elimination—A Narrative Review

**DOI:** 10.1155/jotm/4251955

**Published:** 2026-02-12

**Authors:** Haleh Hanifian, Mehdi Nateghpour

**Affiliations:** ^1^ Department of Medical Parasitology and Mycology, School of Public Health and Institute of Public Health Research, Tehran University of Medical Sciences, Tehran, Iran, tums.ac.ir

**Keywords:** cross-border transmission, elimination, Iran, malaria, surveillance, vector control

## Abstract

**Background:**

Malaria remains a persistent public health concern in Iran, particularly in southeastern regions bordering Afghanistan and Pakistan. Despite substantial progress over recent decades, challenges such as cross‐border transmission, insecticide resistance, and health system disruptions continue to threaten elimination goals.

**Methods:**

This narrative review synthesized evidence from the World Health Organization (WHO) World Malaria Reports, national surveillance summaries, and peer‐reviewed publications indexed in PubMed and Scopus from 2000 to 2025. Emphasis was placed on case trends, intervention coverage, and cross‐border dynamics.

**Results:**

Iran reduced indigenous malaria cases dramatically from thousands in the early 2000s to fewer than 300 annually by the mid‐2010s and subsequently recorded multiple consecutive years with zero indigenous transmission, according to the WHO surveillance reports. Key achievements included integrated vector management, community engagement, and strengthened cross‐border initiatives. However, interruptions during the COVID‐19 pandemic and a resurgence of malaria in 2022, largely associated with imported infections, operational disruptions, and emerging vector threats, highlighted vulnerabilities in elimination‐phase systems. Additional challenges such as insecticide resistance and the spread of *Anopheles stephensi* further complicate the elimination trajectory.

**Conclusion:**

Iran’s experience illustrates the need for adaptive, multisectoral approaches to malaria control in complex socioecological settings. While elimination remains within reach, achieving the WHO certification will require transparent surveillance metrics, reinforce cross‐border collaboration, and sustain political and financial commitment.

## 1. Introduction

Malaria remains one of the most persistent and challenging infectious diseases worldwide, despite decades of intensive control efforts. According to the World Health Organization (WHO), an estimated 263 million malaria cases and 597,000 deaths occurred globally in 2023, predominantly in sub‐Saharan Africa and parts of Asia [1–7]. However, the burden is not limited to these regions. Countries approaching elimination, such as Iran, continue to face challenges in sustaining low transmission levels, preventing resurgence, and managing imported cases [1, 4–9].

Historically, malaria was endemic across many parts of Iran, particularly in the humid southern and southeastern provinces and the Caspian plains, where it remained a widespread cause of morbidity and mortality well into the 20th century [10–14]. Through the National Malaria Control Program (NMCP), substantial progress was achieved: indigenous cases declined from high levels in the early 2000s to fewer than 300 annually by the mid‐2010s [11, 15–19]. According to the WHO surveillance reports, Iran recorded several consecutive years without indigenous malaria transmission, marking an important milestone toward elimination [1, 20, 21].

This success was supported by effective surveillance, integrated vector control, improved case management, community mobilization, and cross‐border initiatives [22–26]. Nevertheless, Iran’s elimination trajectory has not been linear. The COVID‐19 pandemic may have disrupted health systems, diverted resources, and contributed to delays in malaria surveillance and vector control activities [27–32]. Additional challenges, such as continuous cross‐border transmission from Afghanistan and Pakistan, insecticide resistance in Anopheles vectors, and ecological or climatic shifts associated with climate variability and agricultural practices, have further complicated the elimination pathway [25, 32–39].

In 2022, southeastern provinces experienced a resurgence of malaria, revealing vulnerabilities in elimination‐phase systems [10, 32]. Although outbreaks were eventually controlled, this episode underscored the fragility of progress and the urgent need for vigilance, adaptability, and sustained investment in near‐elimination settings [3, 40–42].

Beyond prior overviews, this review synthesizes recent elimination‐phase evidence with emphasis on the 2022 resurgence, programmatic indicators, cross‐border dynamics with Afghanistan and Pakistan, and readiness criteria relevant to the WHO certification and prevention of re‐establishment (PoR) [17, 32, 41, 43–45].

## 2. Methods

This study was conducted as a narrative review aiming to synthesize the best available evidence on malaria trends, elimination progress, and cross‐border transmission in Iran. Given the descriptive nature of the review, the methodology follows commonly accepted standards for narrative synthesis rather than systematic review protocols.

### 2.1. Search Strategy

A multidatabase search was performed in PubMed, Scopus, Web of Science, WHO IRIS, SID, and IranDoc for studies published between January 2000 and May 2025.

Search terms (combined using Boolean operators) included the following: *“malaria,” “Iran,” “elimination,” “border malaria,” “Plasmodium vivax,” “Plasmodium falciparum,” “resurgence,” “Anopheles stephensi,” “vector control,” “surveillance,”* and *“1-2-5 strategy.”*

Gray literature (WHO EMRO briefings, national annual malaria reports, and MoH surveillance data) was included to cover policy‐relevant variables not available in journals.

### 2.2. Inclusion and Exclusion Criteria

Eligible sources included national surveillance reports, the WHO technical documents, and peer‐reviewed articles addressing malaria epidemiology, elimination strategies, vector control, or outbreak investigations in Iran. Nonscientific commentaries, isolated case reports, and studies lacking primary or secondary epidemiological data were excluded. Publications not in English or Persian were excluded unless they contained original data directly relevant to malaria trends in Iran.

### 2.3. Data Extraction and Synthesis

From each included source, information was extracted on malaria incidence, case classification (indigenous versus imported), intervention strategies, vector control activities, and reported outbreak patterns. In cases where discrepancies occurred among sources, priority was given first to the WHO surveillance data, followed by official national reports, and finally, peer‐reviewed publications. Findings were synthesized narratively to allow comparison across heterogeneous data sources and to preserve chronological consistency.

### 2.4. Data Transparency Statement

To enhance transparency, the authors compiled a verified year‐by‐year dataset of malaria case numbers in Iran (2000–2023), extracted directly from the WHO World Malaria Reports, WHO EMRO regional briefings, and national surveillance documents. This dataset includes locally acquired cases (indigenous and introduced) and, where available, imported cases. Because this review is narrative in nature and does not require formal supplementary datasets, the full table is not included in the manuscript; however, it has been prepared in spreadsheet format and is available to the editors upon request for verification.

### 2.5. Use of AI Tools

ChatGPT (OpenAI, GPT‐5) was used solely for minor grammar correction, language refinement, improved clarity of expression, and figure caption editing under direct human supervision. No part of the data extraction, analysis, content generation, or interpretation was performed by AI. All numerical values, citations, and interpretations were manually verified by the authors, who retain full responsibility for the accuracy and integrity of the manuscript.

### 2.6. Limitations

As a narrative review, this study did not employ systematic review protocols such as PRISMA. Although multiple databases and official reports were consulted, some relevant studies may have been missed. Additionally, reliance on routine surveillance data may result in under‐reporting or misclassification, particularly in border provinces. Therefore, findings should be interpreted as a synthesis of the most reliable available evidence rather than an exhaustive systematic assessment.

## 3. Historical Overview of Malaria in Iran

The history of malaria in Iran is deeply rooted, with records of recurrent fevers, locally known as nobeh (periodic fever), appearing in early medical texts and folklore. During the Qajar era (18th‐19th centuries), malaria was a major health challenge, particularly in lowland provinces. By the late 19th century, foreign physicians and travelers documented malaria as one of the most urgent public health concerns in Iran. In 1949, before organized control efforts began, an estimated 40% of the rural population lived in highly endemic areas, and historical reports suggest that malaria accounted for a substantial proportion of deaths in Caspian and Persian Gulf regions [12, 13].

The launch of the NMCP in 1951 marked a turning point, introducing vector control, environmental management, and organized case detection with strong WHO support [12, 13]. Indoor residual spraying (IRS) with DDT, larviciding, and mass drug administration substantially reduced malaria incidence by the 1970s [12, 13]. However, the 1979 revolution and the Iran–Iraq war strained resources and caused setbacks, particularly in southeastern provinces bordering Afghanistan and Pakistan. Despite these disruptions, the control infrastructure remained functional, enabling renewed progress in the 1990s [11–13].

**FIGURE 1:**
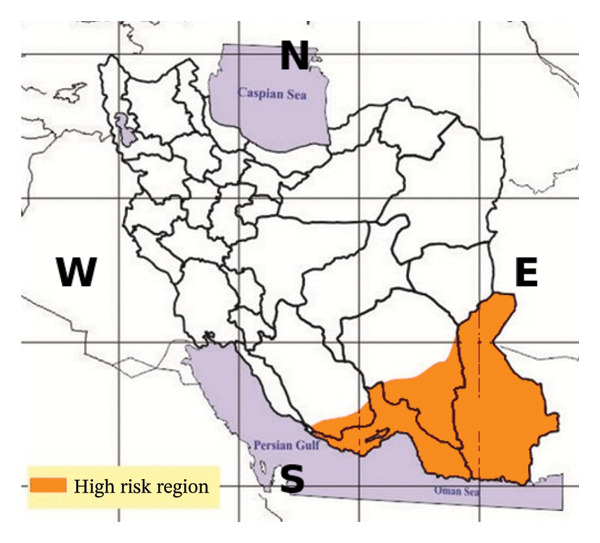
Malaria risk map of Iran highlighting high‐risk regions (orange) mainly in the southeastern provinces, including Sistan and Baluchestan, southern Kerman, and Hormozgan. Adapted from Hanafi‐Bojd et al. [16].

**FIGURE 2:**
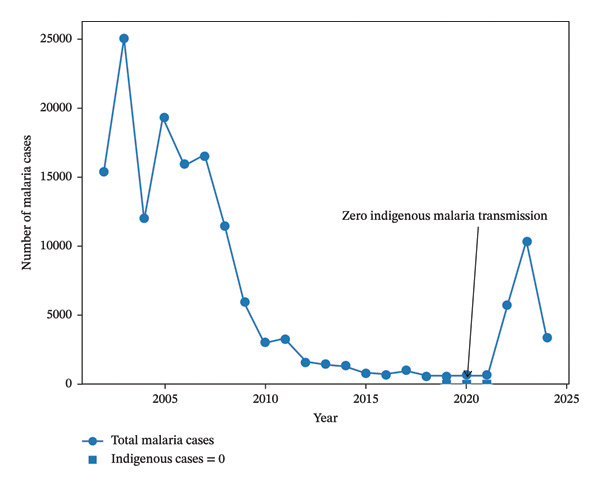
Trends in reported malaria cases in Iran from 2000 to 2024. The solid line represents the total number of confirmed malaria cases (including imported and indigenous infections), based on data extracted from the WHO World Malaria Reports and official national malaria surveillance records.

In the early 2000s, Iran adopted an integrated approach to malaria control, introducing insecticide‐treated nets (ITNs), rapid diagnostic tests (RDTs), active case detection (ACD), and artemisinin‐based combination therapies (ACTs) [11–13, 45]. Investments in human resource training, entomological surveillance, and community participation were prioritized, especially in remote border regions. In 2009, Iran launched a national elimination roadmap aligned with WHO’s global strategy, targeting interruption of local transmission by 2025 [11, 13, 45–48]. Efforts in high‐burden provinces including Hormozgan, Kerman, and Sistan and Baluchestan (Figure 1) included GIS‐based risk stratification and community health worker networks [16, 49, 50]. By the mid‐2010s, indigenous cases had declined to very low levels, and multiple districts reached the pre‐elimination phase. In recent years, Iran recorded several consecutive years without indigenous transmission, positioning the country close to elimination [1, 21, 22]. These trends are illustrated in Figure 2, which summarize reported malaria cases in Iran from 2000 to 2024.

Beyond epidemiological trends, malaria historically shaped livelihoods, agricultural practices, and education disproportionately affecting rural and marginalized communities [12, 14, 49–53]. At the same time, the long history of control reflects resilience: Iranian scientists, physicians, and health workers have built one of the most adaptive malaria elimination programs in the region [13, 45, 46, 54].

In addition to the decline in incidence, programmatic indicators show consistently high IRS coverage with strong community acceptance, and ACD has proven particularly effective, accounting for up to 90% of the cases identified in some border districts. ITN distribution campaigns reached wide coverage although utilization rates were lower than distribution levels [11, 23, 47, 48]. RDT supply chains were disrupted during the COVID‐19 pandemic, and systematic national data on ACT coverage remain limited. Overall, while the control infrastructure is robust, sustaining elimination will require continued high IRS and ACD performance, along with improved monitoring of ITN use, diagnostic availability, and treatment coverage [21, 47, 53] (Table 1).

**TABLE 1:** Major interventions and program indicators in Iran (2008–2014 and program KPIs).

Intervention	Indicator/metric	Years and location	Primary source
Indoor residual spraying (IRS)	Household coverage 96.5%; acceptance 94%	2013‐2014; Sistan and Baluchestan (districts)	Sakeni et al., 2015
Indoor residual spraying (IRS)	Household coverage 85.1%	2008–2011; Sistan and Baluchestan	Nejati et al., 2012
LLIN/ITN	∼80% rural protocol coverage; 3560 nets (2009), 2660 nets (2010) distributed	2009‐2010; Jask, Hormozgan	Fekri et al., 2014
Active case detection (ACD)	Cases detected via ACD: 687 (2006), 879 (2007), 335 (2008), 118 (2009), 87 (2010)	2006–2010; Jask, Hormozgan	Fekri et al., 2014
Share of ACD detections	ACD share ≈90% (Konarak); ≈88% (Nikshahr)	Year(s) per source; Sistan and Baluchestan	Nejati et al., 2012
1–2–5 case management (timeliness KPIs)	Timely notification (1 day), investigation (2 days), foci response (5 days)—definition per the WHO	National policy period; Iran	WHO elimination/certification documents
RDT supply and diagnostics	Narrative reports of stockouts/delays during COVID‐19 (no national quant)	2020‐2021; national	WMR 2023
ACT availability/treatment policy	Policy in place; quantified coverage not published	Policy period; national	National guideline/WHO policy summary

### 3.1. Impact of the COVID‐19 Pandemic

The emergence of the COVID‐19 pandemic in early 2020 posed significant challenges to health systems worldwide, including malaria‐endemic countries such as Iran. In a healthcare system already engaged in malaria elimination, the pandemic likely diverted resources, disrupted operational programs, and strained routine service delivery [54–56]. As efforts shifted toward COVID‐19 control, essential malaria interventions, such as IRS, distribution of ITNs, and ACD, may have been interrupted in high‐risk provinces [17, 21, 32–34, 54, 57].

No quantitative national coverage metrics for IRS, ITN distribution, or RDT availability were reported during this period; published reviews similarly highlight gaps in consolidated program data [21, 22, 32, 34, 55–57]. Therefore, while available evidence suggests that COVID‐19–related disruptions coincided with increased vulnerability to malaria, causal links should be interpreted with caution [21, 22, 35, 54–58].

Border areas adjoining Afghanistan and Pakistan, already difficult to access due to terrain and security conditions, experienced delays in entomological surveillance, case reporting, and vector control operations [59]. Moreover, mobile malaria teams and community health volunteers, who play a critical role in ACD activities in remote villages, were frequently reassigned to COVID‐19 duties, reducing the reach of malaria‐specific services [17, 32, 35, 56–58]. Symptom overlap between COVID‐19 and malaria further complicated diagnosis, as febrile individuals were often reluctant to seek care [54, 58]. In addition, global supply chain disruptions affected the availability of insecticides, diagnostic kits, and antimalarial medicines, potentially undermining preparedness in hotspot areas [21, 22, 32, 58–60].

Reports from the WHO and national studies indicate that the indirect impact of the pandemic on malaria control varied across Iran. While low‐endemic and malaria‐free regions largely maintained surveillance and case management, increases in malaria incidence were reported in southeastern provinces during the early 2020s [17, 21, 36, 59–61]. These patterns highlighted vulnerabilities in elimination programs when faced with large‐scale external crises, particularly in settings dependent on continuous surveillance and rapid response [17, 21, 36, 57, 60, 61].

In response, Iran’s health authorities, in collaboration with the WHO and regional partners, implemented mitigation strategies to sustain malaria services. These included remote training for healthcare workers, revised protocols for fever screening, community‐based malaria testing, and enhanced protective measures for vector control teams [59–62]. Nevertheless, the experience revealed weaknesses in the resilience of elimination‐phase programs during public health emergencies, underscoring the need for stronger contingency planning, more secure supply chains, and closer integration of malaria activities into broader emergency response frameworks [17, 21, 32, 35, 36, 54–62].

### 3.2. The 2022 Malaria Resurgence

After achieving several consecutive years without indigenous malaria, Iran experienced a resurgence in 2022. However, in 2022, the southeastern province of Sistan and Baluchestan reported a resurgence of malaria. According to the WHO’s Eastern Mediterranean Regional Office and national surveillance data, this increase was likely associated with intensified cross‐border transmission, operational disruptions linked to the COVID‐19 pandemic, and emerging insecticide resistance in local Anopheles populations [17, 21, 32, 35, 63–73].

Entomological surveys confirmed the spread of *Anopheles stephensi*, an efficient urban malaria vector, in border and periurban areas, mirroring patterns observed in neighboring countries [20, 23, 62, 73]. Together with rising pyrethroid resistance, this species challenged conventional control tools such as ITNs and IRS. Resistance monitoring data revealed reduced susceptibility in *An. stephensi* and *An. culicifacies*, prompting the use of alternative insecticides such as bendiocarb and pirimiphos‐methyl [48, 62–64, 68, 72–75].

### 3.3. Cross‐Border Dynamics

Cross‐border malaria transmission from Pakistan and Afghanistan has played a critical role in sustaining transmission in southeastern Iran. Political instability and weak healthcare systems in these neighboring countries have fueled migration, with mobile and undocumented populations crossing into endemic districts of Sistan and Baluchestan and Hormozgan [10, 16, 20, 23, 62, 71].

In response, Iran’s Ministry of Health, in collaboration with the WHO and regional partners, intensified control operations. Emergency IRS campaigns were launched, ITN distributions resumed, mobile teams reactivated, and targeted active case detection strengthened in high‐risk villages. Surveillance systems were reinforced, and RDTs distributed to border health posts and mobile clinics to improve detection among migrants and travelers [17, 20, 21, 63, 71, 74].

While these measures improved local preparedness, their long‐term impact has been constrained by inconsistent funding, limited coordination with neighboring countries, and security challenges in border regions. The absence of an institutionalized trilateral monitoring framework (Iran–Afghanistan–Pakistan) further undermines the effectiveness of cross‐border collaboration. These gaps highlight that although Iran has made significant efforts, strengthening cross‐border mechanisms remains essential for sustaining elimination progress and achieving WHO certification [17, 20, 21, 62–65, 71, 74].

Although these interventions successfully contained the outbreak by late 2022, the episode revealed vulnerabilities in Iran’s elimination program. It underscored the need for sustained vigilance, stronger cross‐border collaboration, and adaptive vector control strategies capable of addressing both insecticide resistance and emerging urban malaria threats [20, 36, 62–64, 68, 72–74]. Ultimately, the 2022 resurgence emphasized that without robust surveillance, fortified border health systems, and proactive partnerships with Afghanistan and Pakistan, Iran’s progress toward WHO malaria‐free certification remains at risk [17, 20, 21, 36, 62–65, 71, 74].

### 3.4. Challenges and Future Directions

As Iran advances toward malaria elimination, several persistent and emerging challenges continue to threaten progress. The most critical is cross‐border transmission, which remains the primary source of imported cases. Iran shares porous borders with Pakistan and Afghanistan, both with substantially higher malaria burdens. Continuous migration, trade, and refugee movements introduce new cases and complicate surveillance in southeastern provinces [10, 16, 20, 23, 62].

Another major concern is insecticide resistance in *An. stephensi* and *An. culicifacies*. Reduced susceptibility to pyrethroids undermines the effectiveness of ITNs and IRS. The recent establishment of *An. stephensi* in urban areas increases the risk of outbreaks in densely populated settings [48, 62–64].

Environmental changes—including urban expansion, irrigation, and water management projects—have also modified mosquito habitats, potentially prolonging transmission seasons and expanding risk areas [39, 62]. Meanwhile, the COVID‐19 pandemic revealed weaknesses in malaria elimination infrastructure. Interruptions in surveillance, case management, and vector control during 2020‐2021 underscored the need for contingency planning to sustain services during crises [17, 21, 22, 32, 35].

Looking ahead, Iran should prioritize the following:

• Cross‐border coordination: joint surveillance, synchronized vector control campaigns, and regional health initiatives with Pakistan and Afghanistan [10, 16, 20, 23, 62].
• Resistance management: expanded monitoring, use of alternative insecticides, and piloting of next‐generation vector control tools [48, 62–64].
• Urban malaria preparedness: integration of *An. stephensi* surveillance into municipal systems, mapping breeding sites, and community education [20, 23, 62].
• System resilience: investments to maintain malaria services during pandemics, natural disasters, or political instability [17, 21, 32, 35].
• IVM: regionally tailored strategies supported by data‐driven decision‐making and community participation [16, 20, 23, 75].

The lessons from Iran’s malaria elimination journey, particularly the setbacks during COVID‐19 and the 2022 resurgence, offer valuable guidance for other countries navigating the final, and often most difficult, stage of malaria control.

### 3.5. Path to the WHO Certification

For Iran, malaria elimination is more than a technical milestone; it reflects decades of perseverance, innovation, and resilience. Achieving the WHO certification requires not only four consecutive years without indigenous cases but also strong evidence that local transmission will not be re‐established. Iran had approached this milestone in recent years, but the resurgence in 2022 reset the certification timeline [17, 21, 36].

The WHO certification emphasizes the robustness of surveillance and response systems. Key indicators include implementation of the 1‐2‐5 strategy (case notification within one day, case investigation within two days, and focus response within five days), accuracy of case classification, and the strength of reactive case detection and foci management. Countries must also present a clear prevention‐of‐reestablishment plan [17, 76].

Although Iran has made progress by reinforcing vector control, digitalizing reporting, and training health personnel in border provinces, quantitative assessments of these technical indicators remain limited. No national data have been published on the timeliness of 1‐2‐5 implementation, and systematic evidence on reactive case detection and foci management is scarce. Cross‐border transmission further complicates certification prospects. Despite past achievements, continuous importation from Afghanistan and Pakistan poses a significant threat. The literature provides little systematic data on screening at border points, referral timeliness, or the effectiveness of bilateral collaborations [10, 16, 17].

Taken together, these limitations suggest that while Iran is advancing toward elimination, available evidence is insufficient to fully demonstrate readiness for the WHO certification. Future evaluations will require transparent reporting of surveillance metrics, detailed foci investigations, and measurable outcomes of cross‐border collaboration.

## 4. Conclusion

Malaria elimination is more than a technical milestone; it reflects the broader capacity of a health system to address ecological, climatic, cultural, and political challenges. In many parts of Iran, environmental and socioeconomic conditions have historically sustained transmission, and breaking this cycle requires strategies that go beyond medical interventions to tackle the underlying ecological and climatic drivers of risk [3, 12, 13, 17, 39].

Health system disruptions, whether due to conflict, political instability, or natural disasters, can rapidly reverse progress. Historical precedents, including malaria outbreaks in northern Iran following the dissolution of the Soviet Union in 1991 and the resurgence of cases during the COVID‐19 pandemic, underscore the fragility of malaria control achievements and highlight the necessity for sustained preparedness and contingency planning [3, 10, 12, 17].

Iran’s progress toward elimination demonstrates resilience, scientific capacity, and regional collaboration. However, the 2022 resurgence confirmed that achievements remain vulnerable, particularly in border provinces with sustained importation pressures.

Experiences from Saudi Arabia and Sri Lanka, two countries that achieved the WHO malaria‐free certification, provide useful lessons for Iran. Saudi Arabia sustained compliance with the 1‐2‐5 case management strategy, supported by routine timeliness audits and external reviews [41, 65, 71]. Sri Lanka eliminated malaria in 2012 and was certified malaria‐free in 2016, maintaining rigorous prevention‐of‐reintroduction (POR) systems that integrated entomological surveillance, case classification quality assurance, and rapid foci response. Both countries also institutionalized cross‐border screening and referral protocols through bilateral data‐sharing agreements [44].

For Iran, adapting these practices will require strengthened operating procedures with Afghanistan and Pakistan, systematic reviews of surveillance timeliness, and ring‐fenced POR funding to ensure resilience against resurgence [41, 44, 65, 71, 77].

Looking ahead, Iran’s path to the WHO certification will depend on sustained political commitment, active community engagement, and strengthened cross‐border partnerships. With continued investment and adaptive strategies, the vision of a malaria‐free Iran is achievable and could serve as a regional model for elimination in complex socioecological settings.

## Funding

No funding was received for this manuscript.

## Conflicts of Interest

The authors declare no conflicts of interest.

## Data Availability

Data sharing is not applicable to this article as no datasets were generated or analyzed during the current study.
